# Supplementary material for: Expert consensus on the important chronic non-specific neck pain motor control and segmental exercise and dosage variables: An international e-Delphi study
Source: PLoS One. 2021 Jul 1;16(7):e0253523. doi: 10.1371/journal.pone.0253523 (PMC8248695; doi:10.1371/journal.pone.0253523)
Supplement: S1 Table — Abbreviations: IQR: Interquartile range; %: Percentage agreement–The percentage of experts rating the statement as agree or strongly agree; ✖: round consensus criteria not achieved;✔ round consensus criteria achieved. (PDF) [file pone.0253523.s001.pdf]

S1 Table. Statement consensus rounds 2 and 3

		Round 2				Round 3			
**Statement**	**Exercise Type**	**Median**	**IQR**	**%**	**Consensus**	**Median**	**IQR**	**%**	**Consensus**
**Theme 1**									
It is important that **exercise programmes are a continuum** starting with a focus on restoring or normalising quality of movement (Motor Control Exercise) then **progressing exercise and dosage variables over time** to focus on increasing the motor capacity or outputs of the cervical musculature (Segmental Exercise) *(Expert & Clinical Trial data)*	**NA**	4.00	2.00	69.23	✗	-	-	-	-
It is important that **exercise programmes start with concurrent exercises** to restore or normalise the quality of movement (Motor Control Exercise) **AND** exercises to increase motor capacity or output of the cervical musculature (Segmental Exercise) *(Clinical Trial data)*	**NA**	4.00	1.00	53.85	✗	-	-	-	-
It is important that exercise and dosage variables are **tailored to each patient** *(Expert Opinion data)*	**Motor Control**	5.00	1.00	92.00	✓	5.00	1.00	95.83	✓
	**Segmental**	5.00	0.00	100.00	✓	5.00	0.75	100.00	✓
It is important that exercise and dosage variables are **prescribed to specifically improve patient reported symptoms** (e.g. pain, fear avoidance, low self-efficacy) *(Expert Opinion data)*	**Motor Control**	4.00	1.50	76.00	✓	4.00	0.75	75.00	✓
	**Segmental**	4.00	1.50	76.00	✓	4.00	1.00	66.67	✗
It is important that exercise and dosage variables are **prescribed specifically for each muscle group** (e.g. deep cervical flexors would be different to deep cervical extensors) *(Expert Opinion & Clinical Trial data)*	**Motor Control**	4.00	3.00	69.23	✗	-	-	-	-
	**Segmental**	4.00	3.00	65.38	✗	-	-	-	-
It is important that exercise and dosage variables are **prescribed specifically to change neuromuscular function or motor capacity** (i.e. skill acquisition, strength, power, or endurance) based on assessment findings and patients' functional goals or demands *(Expert Opinion data)*	**Motor Control**	4.00	1.25	76.92	✓	4.00	1.00	79.17	✓
	**Segmental**	4.50	1.00	80.77	✓	4.00	1.00	79.17	✓
It is important that exercise and dosage variables are **rigidly prescribed by exercise professionals** *(Expert Opinion & Clinical Trial data)*	**Motor Control**	2.50	2.00	15.38	✗	-	-	-	-
	**Segmental**	3.00	2.00	15.38	✗	-	-	-	-
It is important that exercise and dosage variables are **prescribed in collaboration with the patient** *(Expert Opinion data)*	**Motor Control**	5.00	1.00	88.46	✓	4.50	1.00	87.50	✓
	**Segmental**	5.00	1.00	96.15	✓	5.00	1.00	95.83	✓
It is important that **patients are provided with a framework within which they can choose** exercise and dosage variables **rather than following a rigid prescription** *(Expert Opinion data)*	**Motor Control**	4.00	2.00	65.38	✗	-	-	-	-
	**Segmental**	4.00	2.00	73.08	✗	-	-	-	-
It is important that **patients self-select** exercise and dosage variables **without guidance from exercise professionals** *(Expert Opinion data)*	**Motor Control**	2.00	1.00	11.54	✗	-	-	-	-
	**Segmental**	2.00	1.00	11.54	✗	-	-	-	-
It is important that exercise and dosage variables are **kept simple** *(Expert Opinion data)*	**Motor Control**	5.00	1.00	92.31	✓	5.00	1.00	95.83	✓
	**Segmental**	5.00	1.00	92.31	✓	5.00	0.75	100.00	✓
It is important that exercise and dosage variables are **acceptable to the patient** *(Expert Opinion data)*	**Motor Control**	5.00	1.00	88.46	✓	5.00	1.00	87.50	✓
	**Segmental**	5.00	1.00	96.15	✓	5.00	1.00	91.67	✓
It is important that exercise and dosage variables result in **exercise being an achievable challenge** *(Expert Opinion data)*	**Motor Control**	5.00	1.00	92.31	✓	5.00	1.00	95.83	✓
	**Segmental**	5.00	1.00	96.15	✓	5.00	1.00	100.00	✓
It is important that **patients consider** exercise and dosage variables **to be realistic** *(Expert Opinion data)*	**Motor Control**	5.00	1.00	92.31	✓	5.00	0.75	95.83	✓
	**Segmental**	5.00	0.25	96.15	✓	5.00	0.00	100.00	✓
It is important that **patients adhere to completing the exercise rather than completing the specific dosage** *(Expert Opinion data)*	**Motor Control**	4.00	1.00	73.08	✓	4.00	1.00	62.50	✗
	**Segmental**	4.00	1.00	73.08	✓	4.00	1.00	66.67	✗
It is important that exercise and dosage variables are **expected to be adhered to** *(Expert Opinion data)*	**Motor Control**	4.00	1.25	76.92	✓	4.00	0.00	79.17	✓
	**Segmental**	4.00	1.00	80.77	✓	4.00	0.75	83.33	✓
It is important that exercise and dosage variables allow **exercise to be completed at work** *(Expert Opinion data)*	**Motor Control**	3.00	1.00	30.77	✗	-	-	-	-
	**Segmental**	3.00	1.25	26.92	✗	-	-	-	-
It is important that exercise and dosage variables allow **exercise to be completed at home** *(Expert Opinion & Clinical Trial data)*	**Motor Control**	4.00	2.00	69.23	✗	-	-	-	-

		Round 2				Round 3			
**Statement**	**Exercise Type**	**Median**	**IQR**	**%**	**Consensus**	**Median**	**IQR**	**%**	**Consensus**
	**Segmental**	4.00	2.00	65.38	✗	-	-	-	-
It is important that exercise and dosage variables are **prescribed based on equipment availability** *(Expert Opinion data)*	**Motor Control**	4.00	1.00	88.46	✓	4.50	1.75	75.00	✗
	**Segmental**	4.00	1.00	92.31	✓	4.50	1.00	79.17	✓
It is important that exercise and dosage variables are **monitored by patients and adapted independently** *(Expert Opinion data)*	**Motor Control**	4.00	1.00	65.38	✓	4.00	0.75	75.00	✓
	**Segmental**	4.00	1.00	73.08	✓	4.00	0.75	79.17	✓
It is important that exercise and dosage variables allow **exercise to be completed in a group setting** *(Expert Opinion data)*	**Motor Control**	3.00	1.00	7.69	✗	-	-	-	-
	**Segmental**	3.00	1.00	12.00	✗	-	-	-	-
It is important that exercise and dosage variables are **changed periodically to prevent boredom** *(Expert Opinion data)*	**Motor Control**	4.00	1.25	53.85	✗	-	-	-	-
	**Segmental**	4.00	2.00	57.69	✗	-	-	-	-
It is important that **patients are educated so that they understand and accept the rationale for the exercise and dosage variables prescribed** *(Expert Opinion data)*	**Motor Control**	-	-	-	-	5.00	0.75	91.67	✓
	**Segmental**	-	-	-	-	5.00	1.00	95.83	✓
It is important that exercise and dosage variables are **prescribed collaboratively with patients within a framework that is sufficient to affect neuromuscular performance** *(Expert Opinion data)*	**Motor Control**	-	-	-	-	5.00	1.00	91.67	✓
	**Segmental**	-	-	-	-	5.00	1.00	91.67	✓
It is important that prescribed exercise and dosage variables are **not time consuming for the patient to complete** *(Expert Opinion data)*	**Motor Control**	-	-	-	-	4.00	1.00	79.17	✓
	**Segmental**	-	-	-	-	4.00	1.00	87.50	✓
It is important that exercise and dosage variables are **adapted for different stages of rehabilitation process** *(Expert Opinion data)*	**Motor Control**	-	-	-	-	5.00	0.00	95.83	✓
	**Segmental**	-	-	-	-	5.00	1.00	100.00	✓
**Theme 2**									
It is important to prescribe the number of training sessions per a specific timeframe (daily or weekly basis) **(Frequency)** *(Expert Opinion & Clinical Trial data)*	**Motor Control**	4.00	1.00	84.62	✓	4.00	1.00	87.50	✓
	**Segmental**	4.00	1.00	92.31	✓	4.00	1.00	95.65	✓
It is important to prescribe the amount of weight moved **(Load)** *(Expert Opinion & Clinical Trial data)*	**Motor Control**	4.00	2.00	65.38	✗	-	-	-	-
	**Segmental**	4.00	1.00	80.77	✓	4.00	1.00	87.50	✓
It is important to prescribe the number of times an exercise is repeated within a given set **(Repetitions)** *(Expert Opinion & Clinical Trial data)*	**Motor Control**	4.00	1.00	84.62	✓	4.00	0.75	79.17	✓
	**Segmental**	4.00	1.00	84.62	✓	4.00	1.00	91.30	✓
It is important to prescribe the number of groups of consecutive repetitions **(Sets)** *(Expert Opinion & Clinical Trial data)*	**Motor Control**	4.00	1.00	88.46	✓	4.00	0.00	79.17	✓
	**Segmental**	4.00	1.00	88.46	✓	4.00	0.75	87.50	✓
It is important to prescribe the time between each repetition where muscle is relaxed **(Rest between repetitions)** *(Expert Opinion & Clinical Trial data)*	**Motor Control**	3.50	1.25	50.00	✗	-	-	-	-
	**Segmental**	3.50	1.25	50.00	✗	-	-	-	-
It is important to prescribe the time between each set where muscle is relaxed **(Rest between sets)** *(Expert Opinion & Clinical Trial data)*	**Motor Control**	4.00	1.00	65.38	✓	4.00	1.00	58.33	✗
	**Segmental**	4.00	1.00	69.23	✓	4.00	1.00	58.33	✗
It is important to prescribe the time between each different exercise where muscle is relaxed **(Rest between different exercises)** *(Clinical Trial data)*	**Motor Control**	3.00	1.00	30.77	✗	-	-	-	-
	**Segmental**	3.00	1.00	29.17	✗	-	-	-	-
It is important to prescribe the total days spent resting in between bouts of exercise **(Recovery days)** *(Expert Opinion data)*	**Motor Control**	-	-	-	-	-	-	-	-
	**Segmental**	4.00	2.00	73.08	✗	-	-	-	-
It is important to prescribe the period exercise training programmes are continued for (either weeks or months) **(Duration of exercise training programme)** *(Expert Opinion & Clinical Trial data)*	**Motor Control**	4.00	0.50	76.92	✓	4.00	1.75	70.83	✗
	**Segmental**	4.00	1.00	84.62	✓	4.00	0.75	79.17	✓
It is important to prescribe the most weight that can be lifted for a defined number of repetitions **(Repetition maximum)** *(Expert Opinion & Clinical Trial data)*	**Motor Control**	-	-	-	-	-	-	-	-
	**Segmental**	4.00	1.25	65.38	✓	3.50	1.00	50.00	✗
It is important to prescribe the subjective measurement of the sense of effort during exercise **(Intensity of effort)** *(Expert Opinion & Clinical Trial data)*	**Motor Control**	4.00	1.00	80.77	✓	4.00	1.00	83.33	✓
	**Segmental**	4.00	1.00	84.62	✓	4.00	1.00	95.83	✓
It is important to prescribe the percentage of maximal force achieved by a muscle **(Intensity of maximal voluntary contraction)** *(Expert Opinion & Clinical Trial data)*	**Motor Control**	3.00	1.00	42.31	✗	-	-	-	-
	**Segmental**	3.00	1.00	46.15	✗	-	-	-	-

		Round 2				Round 3			
**Statement**	**Exercise Type**	**Median**	**IQR**	**%**	**Consensus**	**Median**	**IQR**	**%**	**Consensus**
**Theme 3**									
It is important to prescribe the time taken to complete a single repetition **(Repetition speed)** *(Expert Opinion & Clinical Trial data)*	**Motor Control**	4.00	1.00	56.00	✘	-	-	-	-
	**Segmental**	4.00	1.00	66.67	✔	4.00	1.00	58.33	✘
It is important to prescribe the ratio that defines the time spent in different phases of an exercise (e.g. 4/0/2/0 denotes a 4 second eccentric phase, no break in the transition, a 2 second concentric phase and no rest before the next repetition) **(Cadence/Tempo)** *(Expert Opinion data)*	**Motor Control**	4.00	1.50	56.00	✘	-	-	-	-
	**Segmental**	4.00	1.50	52.00	✘	-	-	-	-
It is important to prescribe the time a muscle is under strain during a set of exercise **(Time under tension)** *(Expert Opinion data)*	**Motor Control**	4.00	1.00	58.33	✘	-	-	-	-
	**Segmental**	4.00	1.00	54.17	✘	-	-	-	-
It is important to prescribe the position an exercise is completed in **(Exercise position)** *(Expert Opinion data)*	**Motor Control**	4.00	1.00	88.00	✔	4.00	1.00	87.50	✔
	**Segmental**	4.00	1.00	92.00	✔	4.00	1.00	91.67	✔
It is important to prescribe the range of movement within which an exercise is completed **(Range of movement)** *(Expert Opinion & Clinical Trial data)*	**Motor Control**	5.00	1.00	88.00	✔	4.00	1.75	75.00	✘
	**Segmental**	5.00	1.00	95.83	✔	4.50	1.00	83.33	✔
It is important to prescribe the surface an exercise is completed on **(Exercise surface)** *(Expert Opinion data)*	**Motor Control**	3.00	1.00	44.00	✘	-	-	-	-
	**Segmental**	3.00	1.75	37.50	✘	-	-	-	-
It is important to prescribe where an individual should direct their attention during exercise **(Attentional focus)** *(Expert Opinion data)*	**Motor Control**	4.00	2.00	64.00	✘	-	-	-	-
	**Segmental**	4.00	1.00	60.00	✔	4.00	1.75	70.83	✘
It is important to prescribe the time of day exercise is completed **(Time of day)** *(Expert Opinion data)*	**Motor Control**	-	-	-	-	-	-	-	-
	**Segmental**	2.00	2.00	4.00	✘	-	-	-	-
It is important to prescribe the direction in which resistance is applied during exercise **(Direction of resistance)** *(Expert Opinion data)*	**Motor Control**	-	-	-	-	-	-	-	-
	**Segmental**	4.00	1.00	92.00	✔	4.00	1.00	87.50	✔
**Theme 4**									
It is important to monitor **technique during exercise** to ensure exercise and dosage variables are appropriate *(Expert Opinion & Clinical Trial data)*	**Motor Control**	4.00	1.00	96.00	✔	5.00	1.00	91.67	✔
	**Segmental**	4.00	1.00	88.00	✔	4.00	1.00	95.83	✔
It is important to monitor **breathing during exercise** to ensure exercise and dosage variables are appropriate *(Expert Opinion & Clinical Trial data)*	**Motor Control**	4.00	1.00	60.00	✔	4.00	2.00	54.55	✘
	**Segmental**	4.00	1.50	60.00	✔	3.00	1.00	43.48	✘
It is important to monitor **muscle group activation during exercise** to ensure exercise and dosage variables are appropriate *(Expert Opinion & Clinical Trial data)*	**Motor Control**	4.00	2.00	64.00	✘	-	-	-	-
	**Segmental**	4.00	1.00	56.00	✘	-	-	-	-
It is important to monitor **patient effort** to ensure exercise and dosage variables are appropriate *(Expert Opinion & Clinical Trial data)*	**Motor Control**	4.00	1.00	80.00	✔	4.00	1.00	91.67	✔
	**Segmental**	4.00	1.00	92.00	✔	5.00	1.00	95.83	✔
It is important to monitor **pain during exercise** to ensure exercise and dosage variables are appropriate *(Expert Opinion & Clinical Trial data)*	**Motor Control**	4.00	1.00	84.00	✔	4.00	1.00	83.33	✔
	**Segmental**	4.00	1.00	84.00	✔	4.00	1.00	83.33	✔
It is important to monitor **pain after exercise** to ensure exercise and dosage variables are appropriate *(Expert Opinion data)*	**Motor Control**	4.00	1.00	88.00	✔	4.50	1.00	87.50	✔
	**Segmental**	4.00	1.00	92.00	✔	4.50	1.00	91.67	✔
It is important to monitor **patient's day to day symptoms** to ensure exercise and dosage variables are appropriate *(Expert Opinion data)*	**Motor Control**	4.00	1.00	68.00	✔	4.00	1.75	70.83	✘
	**Segmental**	4.00	1.00	72.00	✔	4.00	1.50	75.00	✘
It is important to monitor **patient fatigue during exercise** to ensure exercise and dosage variables are appropriate *(Expert Opinion data)*	**Motor Control**	4.00	1.00	80.00	✔	4.00	1.00	87.50	✔
	**Segmental**	4.00	1.00	84.00	✔	4.00	1.00	91.67	✔
It is important to monitor **patient fatigue after exercise** to ensure exercise and dosage variables are appropriate *(Expert Opinion data)*	**Motor Control**	4.00	1.50	72.00	✔	4.00	1.00	70.83	✔
	**Segmental**	4.00	1.00	76.00	✔	4.00	0.00	79.17	✔
It is important to monitor **patient compliance** to ensure exercise and dosage variables are appropriate *(Expert Opinion data)*	**Motor Control**	5.00	1.00	96.00	✔	5.00	1.00	91.67	✔
	**Segmental**	5.00	1.00	100.00	✔	5.00	1.00	95.83	✔
It is important to monitor **delayed onset muscle soreness** to ensure exercise and dosage variables are appropriate *(Expert Opinion data)*	**Motor Control**	3.00	1.00	48.00	✘	-	-	-	-
	**Segmental**	4.00	1.00	54.17	✘	-	-	-	-

Statement	Exercise Type	Round 2 Median	Round 2 IQR	Round 2 %	Round 2 Consensus	Round 3 Median	Round 3 IQR	Round 3 %	Round 3 Consensus
**Theme 5**									
It is important that exercise and dosage variables are **progressively increased over time to make exercise harder** *(Expert Opinion & Clinical Trial data)*	**Motor Control**	4.00	2.00	72.00	✗	-	-	-	-
	**Segmental**	4.00	1.00	92.00	✓	5.00	1.00	100.00	✓
It is important that exercise and dosage variables are **prescribed to ensure exercise is harder than what the patient is used to** *(Expert Opinion data)*	**Motor Control**	3.00	1.00	41.67	✗	-	-	-	-
	**Segmental**	4.00	2.00	64.00	✗	-	-	-	-
**Theme 5 Sub Theme 1**									
It is important to adapt exercise and dosage variables to make exercise harder when **daily pain irritability decreases** *(Expert Opinion data)*	**Motor Control**	4.00	1.00	56.00	✗	-	-	-	-
	**Segmental**	4.00	1.00	68.00	✓	4.00	1.00	58.33	✗
It is important to adapt exercise and dosage variables to make exercise harder when **daily pain severity decreases** *(Expert Opinion data)*	**Motor Control**	4.00	1.00	52.00	✗	-	-	-	-
	**Segmental**	4.00	1.00	60.00	✓	4.00	1.00	54.17	✗
It is important to adapt exercise and dosage variables to make exercise harder when **patients no longer fatigue during exercise** *(Expert Opinion data)*	**Motor Control**	4.00	1.50	68.00	✓	4.00	2.00	73.91	✗
	**Segmental**	4.00	1.00	80.00	✓	4.00	1.00	82.61	✓
It is important to adapt exercise and dosage variables to make exercise harder when **there is consistent good form** *(Expert Opinion data)*	**Motor Control**	4.00	1.50	72.00	✓	4.00	1.00	66.67	✗
	**Segmental**	4.00	2.00	72.00	✗	-	-	-	-
It is important to adapt exercise and dosage variables to make exercise harder when **there is consistent good form within a specific range of movement** *(Expert Opinion & Clinical Trial data)*	**Motor Control**	4.00	1.50	64.00	✓	4.00	1.00	58.33	✗
	**Segmental**	4.00	2.00	64.00	✗	-	-	-	-
It is important to adapt exercise and dosage variables to make exercise harder when **functional goals improve** *(Expert Opinion data)*	**Motor Control**	4.00	1.00	84.00	✓	4.00	1.00	78.26	✓
	**Segmental**	4.00	1.00	88.00	✓	4.00	1.00	86.96	✓
It is important to adapt exercise and dosage variables to make exercise harder when **patient symptoms decrease** *(Expert Opinion data)*	**Motor Control**	3.00	1.00	48.00	✗	-	-	-	-
	**Segmental**	4.00	1.00	64.00	✓	4.00	1.00	70.83	✓
It is important to adapt exercise and dosage variables to make exercise harder when **a patient feels they are ready to do so** *(Expert Opinion data)*	**Motor Control**	4.00	2.00	68.00	✗	-	-	-	-
	**Segmental**	4.00	1.50	76.00	✓	4.00	1.00	87.50	✓
It is important to adapt exercise and dosage variables to make exercise harder when **at a pre-specified time point (e.g. 6 weeks, 8 weeks)** *(Clinical Trial data)*	**Motor Control**	2.00	1.50	24.00	✗	-	-	-	-
	**Segmental**	2.00	2.00	32.00	✗	-	-	-	-
It is important to adapt exercise and dosage variables to make exercise harder **in a structured and consistent way (e.g. every 3 days increase reps by 1)** *(Clinical Trial data)*	**Motor Control**	3.00	2.00	40.00	✗	-	-	-	-
	**Segmental**	4.00	2.00	52.00	✗	-	-	-	-
It is important to adapt exercise and dosage variables to make exercise harder when **pain during exercise decreases** *(Expert Opinion data)*	**Motor Control**	-	-	-	-	4.00	1.00	54.17	✗
	**Segmental**	-	-	-	-	4.00	1.00	62.50	✗
It is important to adapt exercise and dosage variables to make exercise harder when **pain immediately after exercise decreases** *(Expert Opinion data)*	**Motor Control**	-	-	-	-	3.00	1.75	41.67	✗
	**Segmental**	-	-	-	-	3.00	2.00	43.48	✗
It is important to adapt exercise and dosage variables to make exercise harder when **neuromuscular performance has improved based on objective findings** *(Expert Opinion data)*	**Motor Control**	-	-	-	-	4.00	1.00	83.33	✓
	**Segmental**	-	-	-	-	4.00	1.00	79.17	✓
It is important to adapt exercise and dosage variables to make exercise harder when **a patient no longer perceives exercise to be difficult** *(Expert Opinion data)*	**Motor Control**	-	-	-	-	4.00	1.00	91.67	✓
	**Segmental**	-	-	-	-	4.50	1.00	95.83	✓
**Theme 5 Sub Theme 2**									
The number of times an exercise is repeated within a given set **(Repetitions)** is an important variable to manipulate when making exercise harder *(Expert Opinion & Clinical Trial data)*	**Motor Control**	4.00	1.50	76.00	✓	4.00	1.00	79.17	✓
	**Segmental**	4.00	1.00	87.50	✓	4.00	1.00	83.33	✓
The amount of weight moved **(Load)** is an important variable to manipulate when making exercise harder *(Expert Opinion & Clinical Trial data)*	**Motor Control**	-	-	-	-	4.00	2.00	73.91	✗
	**Segmental**	4.00	1.00	96.00	✓	5.00	1.00	100.00	✓
The number of training sessions per a specific timeframe (daily or weekly basis) **(Frequency)** is an important variable to manipulate when making exercise harder *(Expert Opinion & Clinical Trial data)*	**Motor Control**	4.00	2.00	72.00	✗	-	-	-	-
	**Segmental**	4.00	1.00	80.00	✓	4.00	1.00	86.96	✓
	**Motor Control**	4.00	1.50	64.00	✓	4.00	0.00	83.33	✓

		Round 2				Round 3			
**Statement**	**Exercise Type**	**Median**	**IQR**	**%**	**Consensus**	**Median**	**IQR**	**%**	**Consensus**
The number of groups of consecutive repetitions performed in sequence **(Sets)** is an important variable to manipulate when making exercise harder *(Expert Opinion & Clinical Trial data)*	**Segmental**	4.00	1.50	76.00	✔	4.00	0.75	79.17	✔
The subjective measurement of the sense of effort during exercise **(Intensity of effort)** is an important variable to manipulate when making exercise harder *(Expert Opinion & Clinical Trial data)*	**Motor Control**	4.00	2.00	64.00	✘	-	-	-	-
	**Segmental**	5.00	1.00	84.00	✔	4.50	1.00	91.67	✔
The percentage of maximal force achieved by a muscle **(Intensity of maximal voluntary contraction)** is a important variable to manipulate when making exercise harder *(Expert Opinion & Clinical Trial data)*	**Motor Control**	3.00	1.00	40.00	✘	-	-	-	-
	**Segmental**	4.00	1.75	54.17	✘	-	-	-	-
The most weight you can lift for a defined number of repetitions **(Repetition maximum)** is an important variable to manipulate when making exercise harder *(Expert Opinion & Clinical Trial data)*	**Motor Control**	-	-	-	-	-	-	-	-
	**Segmental**	4.00	1.75	62.50	✘	-	-	-	-
The time a muscle is under strain during a set of exercise **(Time under tension)** is an important variable to manipulate when making exercise harder *(Expert Opinion data)*	**Motor Control**	4.00	1.00	56.00	✘	-	-	-	-
	**Segmental**	4.00	2.00	66.67	✘	-	-	-	-
The ratio that defines the time spent in different phases of an exercise **(Cadence/Tempo)** is an important variable to manipulate when making exercise harder *(Expert Opinion data)*	**Motor Control**	4.00	1.00	56.00	✘	-	-	-	-
	**Segmental**	4.00	1.00	52.00	✘	-	-	-	-
The time taken to complete a single repetition **(Repetition speed)** is an important variable to manipulate when making exercise harder *(Expert Opinion data)*	**Motor Control**	4.00	2.00	64.00	✘	-	-	-	-
	**Segmental**	4.00	1.50	72.00	✔	3.50	1.00	50.00	✘
Adding or increasing the length of isometric holds at inner or outer range **(Isometric holds)** is an important variable to manipulate when making exercise harder *(Expert Opinion & Clinical Trial data)*	**Motor Control**	4.00	1.50	56.00	✘	-	-	-	-
	**Segmental**	4.00	1.00	60.00	✔	3.00	1.00	45.83	✘
The range of movement an exercise is completed within **(Range of movement)** is an important variable to manipulate when making exercise harder *(Expert Opinion & Clinical Trial data)*	**Motor Control**	4.00	1.00	80.00	✔	4.00	0.75	75.00	✔
	**Segmental**	4.00	1.00	88.00	✔	4.00	1.00	91.67	✔
The position an exercise is completed in **(Exercise position)** is an important variable to manipulate when making exercise harder *(Expert Opinion data)*	**Motor Control**	4.00	2.00	60.00	✘	-	-	-	-
	**Segmental**	4.00	1.50	76.00	✔	4.00	0.00	83.33	✔
It is important that **the exercise or dosage variable most pertinent to a patient's functional activity** is manipulated when making exercise harder *(Expert Opinion data)*	**Motor Control**	-	-	-	-	5.00	1.00	91.67	✔
	**Segmental**	-	-	-	-	5.00	1.00	95.65	✔

Abbreviations: IQR: Interquartile range; %: Percentage agreement – The percentage of experts rating the statement as agree or strongly agree; ✘*: round consensus criteria not achieved;* ✔ *round consensus criteria achieved*
