# Supplementary material for: Expert consensus on the important chronic non-specific neck pain motor control and segmental exercise and dosage variables: An international e-Delphi study
Source: PLoS One. 2021 Jul 1;16(7):e0253523. doi: 10.1371/journal.pone.0253523 (PMC8248695; doi:10.1371/journal.pone.0253523)
Supplement: Attachment — Submitted filename: PONE-D-21-00717_reviewer opinion.docx [file pone.0253523.s003.docx]

Overall “accept pending revisions”

- The idea is great, it has good feasibility and it is worthy to be investigated. It is directional toward finding the best treatment for non-specific neck pain regarding type, dose of exercise. Its value is situated in making an international consensus that will collect the opinions of many experts all over the world.

**Abstract**

Background: I see it is better to start your abstract with non-specific neck pain and not with neck pain generally.

Conclusion: "Important exercise and dosage variables highlight a complexity of exercise prescription not found in clinical trials". I think this conclusion is not accurate enough. Some papers describe the dosage variables that highlight the complexity of exercise. This is also referred to in the systematic reviews concerned with exercise therapy on CNSNP.

**Introduction**

- Line 35: What is meant by "effect size" here?
- Line 37: What about manipulating exercise type?
- Line 38- 46: I see that exercise dose is not clarified enough in the introduction section.
- Lines 57-77: Why you neglected the pillar part of exercise and the upper limb?

**Aim of the study**

- I think the difference between the two purposes you put here. The first purpose is to have expert opinion on exercises and the other is to gain consensus, and I think the consensus will be reached based on the opinion of the expert too. Please make this clear.

**Methods**

Design

- Line 85: The abbreviation (CREDES) is not clear.

Participants

- Line 95: How many academic and exercise professional expert were recruited to participate in the study?

Round 1

- Line 119: Who has given the overview of exercise to the experts?
- Lines 126- 128: "Previous trial data were collected concurrently during round 1 to allow participants to provide their expert opinion without influence from the literature, reducing experimenter bias.[" What sort of data were provided to the experts and how this data may be influenced if they adapt it from the literature, and how they will give their opinion without returning to the literature and exploring the studied included the exercises used to treat CNSNP?

Round 2

- Line 147: How the thematic analysis of the qualitative data was conducted?

Patient and public involvement

- Line 181: What is meant by public involvement?
- Line 183: What does this abbreviation (GRIPP2-SF) indicate?

Table 1

- How patient can review a systematic review i.e how can he understand the critical appraisal and the conclusions of RCTs? Did they received patient education during having their treatment sessions?

**Discussion**

- Line 275: Did you investigate the adherence to exercise or the researchers composed a statement regarding this? And if not, please mention why?
- Line 317: What is meant by volume of exercise?
- Line 327: Please revise the font of this title.

**Research and clinical implications**

- Line 353: What the abbreviation PPI is for?

**Conclusion**

- Line 364: Exercise position is a new variable mentioned for the first time here.
