# Supplementary material for: Expert consensus on the important chronic non-specific neck pain motor control and segmental exercise and dosage variables: An international e-Delphi study
Source: PLoS One. 2021 Jul 1;16(7):e0253523. doi: 10.1371/journal.pone.0253523 (PMC8248695; doi:10.1371/journal.pone.0253523)
Supplement: Attachment — Submitted filename: Response to Reviewers.docx [file pone.0253523.s004.docx]

We would like to thank all the reviewers and editors who have taken the time in reviewing our manuscript. We have responded to each of the comments below and identified how we have updated the manuscript.

Jonathan Price

Musculoskeletal Physiotherapy Services Birmingham Community Healthcare NHS Foundation Trust, Birmingham, UK. E-mail: Jonathan.price2@nhs.net

| Editor Comments | | | |
| --- | --- | --- | --- |
|  | Comments | | Response |
| 1 | Please ensure that your manuscript meets PLOS ONE's style requirements, including those for file naming. The PLOS ONE style templates can be found at  <https://journals.plos.org/plosone/s/file?id=wjVg/PLOSOne_formatting_sample_main_body.pdf> and  [https://journals.plos.org/plosone/s/file?id=ba62/PLOSOne_formatting_sample_title_authors_affiliations.pdf](about:blank) | | Thank you. We have updated the manuscript to reflect your style requirements |
| 2 | Please provide additional details regarding participant consent. In the ethics statement in the Methods and online submission information, please ensure that you have specified what type you obtained (for instance, written or verbal, and if verbal, how it was documented and witnessed). If your study included minors, state whether you obtained consent from parents or guardians. If the need for consent was waived by the ethics committee, please include this information. | | Thank you. We have updated the manuscript to read  *“Written informed consent was received from all participants before completing any questionnaires. The consent process and each round was conducted electronically and anonymously using REDCap, a secure web application for building and managing online surveys”* **Lines 115-118** |
| 3 | Please correct your reference to "p=0.000" to "p<0.001" or as similarly appropriate, as p values cannot equal zero | | Thank you. We have updated Table 3 to read <0.001 rather than 0.000 |
| 4 | We note that Figure 1 includes an image of a  participant in the study.  As per the PLOS ONE policy (<http://journals.plos.org/plosone/s/submission-guidelines#loc-human-subjects-research>) on papers that include identifying, or potentially identifying, information, the individual(s) or parent(s)/guardian(s) must be informed of the terms of the PLOS open-access (CC-BY) license and provide specific permission for publication of these details under the terms of this license. Please download the Consent Form for Publication in a PLOS Journal (<http://journals.plos.org/plosone/s/file?id=8ce6/plos-consent-form-english.pdf>). The signed consent form should not be submitted with the manuscript, but should be securely filed in the individual's case notes. Please amend the methods section and ethics statement of the manuscript to explicitly state that the patient/participant has provided consent for publication: “The individual in this manuscript has given written informed consent (as outlined in PLOS consent form) to publish these case details”.  If you are unable to obtain consent from the subject of the photograph, you will need to remove the figure and any other textual identifying information or case descriptions for this individual. | | Thank you. The person in this image is the lead author. Therefore we have updated the methods section to read  *“The individual in Fig 1 has given written informed consent (as outlined in PLOS consent form) to publish their image.”* **Lines 113-115** |
| **Reviewer 1** | | | |
| 1 | | Have the authors made all data underlying the findings in their manuscript fully available?  The [PLOS Data policy](http://www.plosone.org/static/policies.action#sharing) requires authors to make all data underlying the findings described in their manuscript fully available without restriction, with rare exception (please refer to the Data Availability Statement in the manuscript PDF file). The data should be provided as part of the manuscript or its supporting information, or deposited to a public repository. For example, in addition to summary statistics, the data points behind means, medians and variance measures should be available. If there are restrictions on publicly sharing data—e.g. participant privacy or use of data from a third party—those must be specified. | Thank you for highlighting this oversight. We have now added an anonymised dataset as a supplementary file (S2 Dataset) |
| 2 | | More attention to make clinical practice a better part of the methods of scientific research is extremely important to me in order to better translate the results found into our practice (EBP). Your research makes an important contribution to this.  I am happy to read that experts believe that exercises should match with the patient. Unfortunately, this does not appear to be the case with interventions in general in patients with non-specific neck pain. RCTs investigate specific interventions such as exercises in a heterogeneous mismatched population as my own research showed. (The clinical reasoning process in randomized clinical trials with patients with non-specific neck pain is incomplete: A systematic review.)  Because clinical reasoning in scientific research is close to my heart, I have given my additional opinion here and there. I don't expect you to do anything with that. | Thank you for your kind words. We agree with all your comments and suggestions. We have been selective with which suggestions to incorporate to reduce the manuscript length. Our edits based on your suggestions are below. |
| 3 | | Introduction  What remains unclear is what the exercises are for. On line 39 you talk about physiological constructs such as strength and endurance. Similarly on line 52 the construct spinal function. However, on lines 41 and 42 you speak of neck pain exercises with the underlying construct exercise-induced hypoalgesia (ref18).  So what I wonder is whether the purpose of the exercise is to improve more physiological constructs as you explain from line 52 or whether the exercise should not improve a spinal function as long as it leads to exercise-induced hypoalgesia.  This distinction seems important to me since for the first group the patient must have a function limitation, such as for example a reduced motor control or endurance or strength, while the second group of “Hypoalgesia” only needs to have pain. This distinction may in turn influence dosage variables since it seems important that the intervention is appropriate for the right group of patients. (Therapeutic validity and effectiveness of preoperative exercise on functional recovery after joint replacement: a systematic review and meta-analysis. Hoogeboom TJ, Oosting E, Vriezekolk JE, Veenhof C, Siemonsma PC, de Bie RA, van den Ende CH, van Meeteren NL.PLoS One. 2012;7(5):e38031. doi: 10.1371/journal.pone.0038031)  Therefore, with regard to the goals of the study it remains unclear to me whether the purpose of the exercises were improving co-ordination (motor control) and improving the ability of the neck to produce, transfer and absorb force (segmental exercise) (lines 52-55) after which, if this improved, pain reduction may occur or was the purpose of the exercises pain reduction regardless of whether a function improved or both. Please clarify the primary purpose of the exercises. | Thank you. You raise an interesting point. We agree that the mechanisms by which exercise results in pain relief are unclear or various. To reflect your point we have updated the manuscript  *“Although the mechanisms by which exercise interventions improve pain or disability are unclear (e.g. exercise induced hypoalgesia, secondary to improvements in spinal function, secondary to improvements in physiological outcomes) systematic review findings demonstrate that the largest reduction in pain (moderate to very large short-term effects) were found for exercise training programmes combining both motor control and segmental exercises.[24]”* **Lines 82-87** |
| 4 | | The method is clearly described. The use of text boxes gives this study qualitatively more depth than a Delphi study in which one only scores on consensus. | Thank you. We see this as a strength of our study and pleased you found it useful. |
| 5 | | It is a good thing that patients (page 18 item 4) also indicate that the intervention should be tailored to each person. I translate that if the exercise should aim to improve the present disability. Here, too, the purpose of the exercise appears to be important. | Thank you. We now think we have clarified this based on your previous recommendations.  *“Although the mechanisms by which exercise interventions improve pain or disability are unclear (e.g. exercise induced hypoalgesia, secondary to improvements in spinal function, secondary to improvements in physiological outcomes) systematic review findings demonstrate that the largest reduction in pain (moderate to very large short-term effects) were found for exercise training programmes combining both motor control and segmental exercises.[24]”* **Lines 82-87** |
| 6 | | Since I am not from the UK I do not understand what is meant by band 5 etcetera at work grade. | Thank you for highlighting that this system is unfamiliar to an international audience. We have included a textual description for each banding.  Band 5/Junior  Band 6/Specialist  Band 7/Advanced  Band 8/Extended Scope or Consultant |
| 7 | | Line 209 Table 3 and line 222 again a table 3. Would you please correct this? | Thank you. This has been updated |
| 8 | | Line 221 A result of round 3 is mentioned in round 2. | Thank you. We have reworded this sentence to make it clear that is a summary of the final statements from round 2.  *“A total of 61 statements (46 motor control; 59 segmental) were taken into Round 3”* **Lines 263-264** |
| 9 | | Discussion Line 246 I would be reluctant to consider the variables about which there is consensus as the truth. Further research should show whether the variables found really matter, I think. Although I think you are right ... (A Delphi Study: Defining a Unicorn. Weisman A, Meakins A, Rotem-Lehrer N.Pain Med. 2018 Jun 1;19(6):1295. doi: 10.1093/pm/pnx327) | Thank you. We agree that expert consensus is a low form of evidence and that future research could change recommendations. As such we have rephrased the statement to reflect this  *“Researchers and clinicians should consider using these themes and statements to guide the prescription of CNSNP exercise in future research and clinical practice.”* **Line 288-290** |
| 10 | | Line 260 You stated: “However, the lack of effect maybe due to inadequacies in the clinical reasoning model used to identify appropriate treatment”. This may be, however, they assess the effect with PROMs without first determining whether the intervention has improved the impairment. For example their impairment “reduced cervical flexibility” remains unclear whether the cervical flexibility has been improved. Improving flexibility alone does not necessarily lead to an experienced improvement measured with an NPRS or NDI. They did not use the correct outcome measure measuring the primary goal of the intervention. | Thank you for the suggestion. We have updated the manuscript to reflect this  *“While other spinal research demonstrates the benefits of individualised treatment,[63] Svedmark et al., 2016 found no difference in pain and disability between an individualised exercise programme tailored to neuromuscular dysfunction versus a non-individualised exercise programme.[64] Although this could suggest individualised exercise may not be beneficial the study did not evaluate the effect of the exercise interventions on the neuromuscular dysfunction on which is was based. Therefore, it is unknown whether there was no difference in pain or disability because of no improvement in the neuromuscular dysfunction the exercise programme intended to improve or because this degree of individualisation is not beneficial.”* **Lines 304-312** |
| 11 | | Ah on line 325 you will discuss the use of outcome measures. I think we give exercises to improve physiological variables. It is therefore important to first determine whether a physiological variable has improved before it can be expected that the patient will experience improved thanks to the improved physiological variable ... In this way we can gain insight into whether the patient has improved thanks to or despite the exercises. That is why, like you, I still believe that interventions should be individualized. | Thank you for your comments. Yes we believe exercise should be individualised to meet patients needs. Unfortunately, as you have highlighted in your own systematic review, this is often not the case in neck pain research. |
| 12 | | Line 283 I agree with your statement on protocol driven. In addition, exercises are investigated in RCTs in heterogeneous populations without it being clear whether the patients have the limitation that matches the exercise. |  |
| 13 | | Kendall's Coefficient of Concordance measures inter-expert agreement across multiple statements. Despite the significance, this overall score remains fair to moderate agreement. Can you discuss in the discussion that the concordance is fair to moderate, but the percentage agreement in table 4 is high. There seems to be a discrepancy here. | Thank you for highlighting this. We have expanded upon this point in the manuscript:  *“Participants open text responses identified that the importance of exercise and dosage variables may differ depending on patient characteristics. This variability between patients is further supported by the slight to fair inter-expert agreement (W=0.142 to 0.311), despite achieving statistical significance (p<0.05).”* **Lines 298-300** |
| **Reviewer 2** | | | |
| 1 | | Dear author(s). I appreciate the work you conducted and produced in the submitted article in PLOS ONE. It is a valuable work and needs minor corrections to be complete. Overall “accept pending revisions”  − The idea is great, it has good feasibility and it is worthy to be investigated. It is directional toward finding the best treatment for non-specific neck pain regarding type, dose of exercise. Its value is situated in making an international consensus that will collect the opinions of many experts all over the world. | Thank you for your positive comments regarding our work. The comments and suggested edits below will provide a greater clarity to the manuscript and improve the quality of our work. |
| 2 | | Background: I see it is better to start your abstract with non-specific neck pain and not with neck pain generally. | Thank you. We have updated the abstract to read  *“Chronic non-specific neck pain is highly prevalent, resulting in significant disability.”* **Lines 22-23** |
| 3 | | Conclusion: "Important exercise and dosage variables highlight a complexity of exercise prescription not found in clinical trials". I think this conclusion is not accurate enough. Some papers describe the dosage variables that highlight the complexity of exercise. This is also referred to in the systematic reviews concerned with exercise therapy on CNSNP. | Thank you. We have clarified this point in the abstract which now reads  *“Multiple exercise and dosage variables are important, resulting in complex and individualised exercise prescription not found in clinical trials.”* **Lines 48-49** |
| 4 | | Introduction - Line 35: What is meant by "effect size" here? | Thank you for highlighting this. We have expanded this point to aid in clarity  *“Although exercise is the mainstay treatment for neck pain, there is still considerable uncertainty over the optimal content or delivery of exercise to maximise the effects of pain or disability.[3-5] In medicine, a dose-response relationship exists,[6, 7] and if considering exercise as medicine, effects on pain and disability can be optimised by manipulating exercise dosage (sets, reps, loads, frequency etc.).”* **Lines 56-61** |
| 5 | | - Line 37: What about manipulating exercise type? | Thank you. We have updated the manuscript to reflect these two points.  *“In medicine, a dose-response relationship exists,[6, 7] and if considering exercise as medicine, effects on pain and disability can be optimised by manipulating exercise dosage (sets, reps, loads, frequency etc.). Precision prescription of dosage and other variables such as exercise type, speed or order increases the effectiveness of achieving physiological outcomes of hypertrophy, strength, power, endurance in healthy populations.”* **Lines 58-64** |
| 6 | | - Line 38- 46: I see that exercise dose is not clarified enough in the introduction section. |  |
| 7 | | - Lines 57-77: Why you neglected the pillar part of exercise and the upper limb? | Thank you. The study focused on motor control and segmental exercises as this combination of exercises was found in a systematic review to have the largest effects on pain.  We have helped to clarify this point by updated the manuscript with the following  *“…systematic review findings demonstrate that the largest effects on CNSNP (moderate to very large short-term effects) were found for exercise training programmes combining both motor control and segmental exercises.”* **Lines 85-87**  *“However, evaluation of optimal motor control and segmental exercise and dosage variables was limited by inconsistent and poor intervention reporting; a finding not limited to CNSNP trials”* **Lines 88-90**  *“Consensus on the important motor control and segmental exercise and dosage variables would strengthen the evidence, informing precision rehabilitation, therefore improving CNSNP.”* **Lines 100-102** |
| 8 | | Aim of the study - I think the difference between the two purposes you put here. The first purpose is to have expert opinion on exercises and the other is to gain consensus, and I think the consensus will be reached based on the opinion of the expert too. Please make this clear. | Thank you.  We have updated the second aim to clarify the consensus process was conducted with experts   1. *“To gain expert opinion on the important motor control and segmental exercise and dosage variables for CNSNP treatment.”* 2. *“To conduct a systematic process with experts to gain consensus on the important motor control and segmental exercise and dosage variables identified in Aim 1”*   **Lines 104-107** |
| 9 | | Design - Line 85: The abbreviation (CREDES) is not clear. | Thank you. We have updated the capitalisation to make this clearer.  *“Guidance on Conducting and Reporting Delphi Studies (CREDES)”* **Lines 111-112** |
| 10 | | Participants - Line 95: How many academic and exercise professional expert were recruited to participate in the study? | Thank you.  51 academic and 10 exercise professionals were identified and contacted by the lead author. **Lines 229-230**  Following consent 3 participants identified as academic experts, 15 identified as clinical experts, and 16 identified as having both academic and clinical expertise. **Table 1**  To make this easier for the reader we have removed the term exercise professional expert from the methods and replaced with “Clinical Expert” to consistency to provide consistency between the text and Table 1. |
| 11 | | - Line 119: Who has given the overview of exercise to the experts? | Thank you for highlighting this. We have updated the manuscript to clarify this point  *“Before completing the questionnaire, the study team provided participants with a standardised summary of the systematic review results informing the Delphi study and with definitions and examples of motor control and segmental exercise.”* **Lines 149-152** |
| 12 | | - Lines 126- 128: "Previous trial data were collected concurrently during round 1 to allow participants to provide their expert opinion without influence from the literature, reducing experimenter bias.[" What sort of data were provided to the experts and how this data may be influenced if they adapt it from the literature, and how they will give their opinion without returning to the literature and exploring the studied included the exercises used to treat CNSNP? | Thank you for identifying this. We have updated the manuscript  *“During round 1 the study team (J.P./V.T.) also extracted exercise and dosage data from clinical trials describing motor control and segmental exercise cited in a recent systematic review.[24] Data was extracted independently and mapped to TIDieR/CERT. [31, 32] Previous trial data were collected concurrently during round 1 and combined with the data collected from experts before being analysed and presented back to the experts in round 2. Collecting clinical trial data and expert opinion data separately in round 1 improved content validity for future rounds.[47, 50, 51]”* **Lines 156 – 162** |
| 13 | | - Line 147: How the thematic analysis of the qualitative data was conducted | Thank you. We have expanded the methods section to provide further details  *“Data was organised by participant as recommended when conducting thematic analysis on questionnaire responses.^[84]^ Following a period of data familiarisation, data was coded iteratively. Codes were organised to develop candidate themes with central organising concepts both inductively, and then deductively informed by TIDieR/CERT. Candidate themes were edited to develop full themes by reviewing the codes and original questionnaire responses. Codes within themes were converted to statements.”* **Lines 164-170** |
| 14 | | Patient and public involvement - Line 181: What is meant by public involvement? | Thank you. Within the UK it is now common practice to involve patients and the public throughout all stages of research (priority setting, developing research questions, study design, study conduct, analysis of results, dissemination). Patients/Public are not research participants but are thought of as part of the study team or researchers. The website <https://www.invo.org.uk/> provides further details around the benefits of Patient and Public Involvement (PPI) in research.  We have updated the manuscript and Table. We have added further text and edited Table 5 to clarify the purpose of PPI for international audiences and make clear they were not part of the consensus process. We have responded to your specific comments regarding PPI below.  *“Patient and public involvement (PPI) is defined by the National Institute for Health Research as research being carried out ‘with’ or ‘by’ patients or the public, rather than ‘to’, ‘about’ or ‘for’ them.”* **Lines 223-225** |
| 15 | | - Line 183: What does this abbreviation (GRIPP2-SF) indicate? | Thank you. We have added full text for the abbreviation  *“PPI was integral to this study through the SSG and their impact on the study is reported using the Guidance for Reporting Involvement of Patients and the Public 2 Short Form (GRIPP2-SF)”* **Lines 225-227** |
| 16 | | Table 1 - How patient can review a systematic review i.e how can he understand the critical appraisal and the conclusions of RCTs? Did they received patient education during having their treatment sessions? | Thank you. The systematic review findings were summarised by the study team and presented using lay language. Table 5 has been updated to reflect this.   1. *“Systematic review findings were summarised and presented to the patients (and SSG) using lay language by the study team for comments”* |
| 17 | | - Line 275: Did you investigate the adherence to exercise or the researchers composed a statement regarding this? And if not, please mention why? | Thank you. A statement relating to adherence was included in Theme 1 Statement 7 which was constructed by expert opinion  *“Variables are expected to be adhered to^*”^* |
| 18 | | - Line 317: What is meant by volume of exercise? | Thank you, we have updated the manuscript  *“in volume (how much exercise is done), rather than frequency”* **Lines 367-368** |
| 19 | | - Line 327: Please revise the font of this title. | Thank you. This has been updated to be consistent with other headings. |
| 20 | | Research and clinical implications - Line 353: What the abbreviation PPI is for? | Thank you. The definition of PPI is provided on **Line 223** |
| 21 | | Conclusion - Line 364: Exercise position is a new variable mentioned for the first time here. | Thank you. Exercise position was highlighted in the discussion section on **Lines 358 & 361** |
| **Reviewer 3** | | | |
| 1 | | The authors have attempted to establish expert consensus regarding optimal exercise and dosage variables for exercises prescribed to chronic non-specific neck pain patients. However, the methods and procedure section are not clearly explained to recommend the manuscript in its current format for publication. | Thank you for your comments and suggested edits below which will provide a greater clarity to the manuscript and improve the quality of our work. |
| 2 | | The authors may consider referring to other studies to develop background for their hypothesis. What’s the difference between objectives 1 and 2?. | Thank you for this comment.  Aim 1 was to gain expert opinion – i.e. to collect data on what variables experts consider important when prescribing exercise  Aim 2 was to conduct a systematic process in an attempt to gain consensus on the important variables from Aim 1. We may or may not have achieved consensus, hence the wording reflecting the systematic process.  We have updated the text to provide this clarity   1. *“To gain expert opinion on the important motor control and segmental exercise and dosage variables for CNSNP treatment.”* 2. *“To conduct a systematic process with experts to gain consensus on the important motor control and segmental exercise and dosage variables identified in Aim 1”*   **Lines 104-107** |
| 3 | | Methods: • It’s not clear how the responses to questionnaires were collected. | Thank you for highlighting this. We have added the following statement  *“The consent process and each round was conducted electronically and anonymously using REDCap, a secure web application for building and managing online surveys.”* ***Lines 116-118*** |
| 4 | | • It’s not clear what the authors mean by ‘explain important exercise and dosage variables for each exercise type when treating CNSNP’ - please define each exercise type. | Thank you. The manuscript has been updated to read  *“…explain important exercise and dosage variables for both motor control and segmental exercises when treating CNSNP…”* **Lines 153-154** |
| 5 | | • Previous trial data were collected concurrently during round 1 to allow participants to provide their expert opinion without influence from the literature – How and from where were this data collected? | Thank you. We have updated the manuscript to clarify this point.  *“During round 1 the study team (J.P./V.T.) also extracted exercise and dosage data from clinical trials describing motor control and segmental exercise cited in a recent systematic review.[24] Data was extracted independently and mapped to TIDieR/CERT. [31, 32] Previous trial data were collected concurrently during round 1 and combined with the data collected from experts before being analysed and presented back to the experts in round 2. Collecting clinical trial data and expert opinion data separately in round 1 improved content validity for future rounds.[47, 50, 51]”* **Lines 156-162** |
| 6 | | • Participants were provided with an overview of the systematic review results- provide citation to the SR | Thank you. We have now added the citation. |
| 7 | | • Isn’t it a common practice to rate their agreement with statements and collect feedback on theme/statement generation on round 1 in Delphi studies using a Likert scale? | Thank you. In the originally described Delphi method, experts are asked for their opinions in Round 1. The Dephi method has been adapted over the years into various forms. A “Modified” Delphi utilises a methodology whereby experts rate items in Round 1 generated through the literature. We have used the original Delphi method and hence described as “Delphi”.  Keeney, S; Hanson, F; McKenna, H (2011) The Delphi Technique in Nursing and Health Research. Wiley-Blackwell |
| 8 | | • What were the criteria to recruit patients in SSG and how were they recruited? Don’t the authors think having the patients with different perceptions of the experts with different educational, professional and cultural backgrounds will affect the outcome? Were the expert members aware of the involvement of patients in the study steering group? Were any statements added based on the recommendation of SSG? If yes, why no description of that has been incorporated in the Delphi procedure section? | Thank you. Within the UK it is now common practice to involve patients and the public throughout all stages of research (priority setting, developing research questions, study design, study conduct, analysis of results, dissemination). Patients/Public are not research participants but are thought of as part of the study team or researchers. The website <https://www.invo.org.uk/> provides further details around the benefits of Patient and Public Involvement (PPI) in research.  We have updated the manuscript and Table We have added further text and edited Table 5 to clarify the purpose of PPI for international audiences and make clear they were not part of the consensus process. We have responded to your specific comments regarding PPI below.  Yes, experts were aware that a SSG provided study oversight.  All statements were derived from either expert opinion or clinical trial data. The SSG agreed on the wording of statements or their inclusion/removal. Statements were not generated from the SSG. We have included the following statement to provide further clarity  *“Members of the SSG who were not co-authors did not have access to raw data or were able to influence the study process. Feedback and changes suggested by the SSG were agreed between the study co-authors before implementation.”* **Lines 218-220** |
| 9 | | • Please clarify – what do the authors mean by ‘Patient representatives suggested the use of “exercise training program” rather than resistance training to collectively describe the exercises described in this study and the systematic review from which this study is based’. | Thank you. We have updated Table 5 to clarify this point   1. *“Original drafts of the systematic review informing this study used the term “Resistance Training” to collectively describe the exercises included in the Delphi study. When systematic review findings were presented to the patient representatives (Method, Point 1) they recommended the term “exercise training programme” rather than resistance training. Patients felt resistance training had a strong association with exercises performed in a gym or fitness center and believed exercise training programme was more appropriate for exercise that could be conducted in any location (i.e. home, gym, clinic). As a result of our PPI, the term exercise training programme has been used in this manuscript.”* |
| 10 | | • The point 5- Patient representatives highlighted their importance of being able to self-regulate exercise is not clear;- during which phase (rounds) of Delphi were these themes developed? | Thank you. We have updated Table 5 to clarify this point with the following text   5. *“Upon completion of the Delphi study, the results were presented to the patient representatives. Patient representatives highlighted their importance of being able to self-regulate exercise. Although this concept achieved consensus, the statement agreement was lower than other statements, suggesting expert and patient opinions on important components of exercise prescription may differ. Patients encouraged future research to evaluate patients’ needs and desires when prescribing exercise as these maybe different to clinicians/experts. As a result of our PPI, we intend to conduct research to investigate the needs of patients that exercise must satisfy, and to develop an exercise programme where patients can self-regulate and monitor exercise and dosage variables.”* |
| 11 | | • There are so many decisions that seem to be supported by the patients. Considering only 2 patients were present in steering committee, how was it achieved? Can this be called a consensus/ opinion? | Thank you for raising this point. The GRIPP2-SF outlines the patient and public involvement in this study as part of the Study Steering Group. The patients included in this were **not** research participants or part of the consensus process. We have clarified this point with the following text  *“Patient representatives were not involved in the consensus procedures.”* |
| 12 | | • At exactly which part of the Delphi interview, did the patient steering group members felt patients should be made aware that clinicians may not have all the answers and there are gaps in the literature. | Thank you. We have updated Table 5 to clarify this point   6. *“Upon completion of the Delphi study, patient representatives also provided recommendations as to how the findings of this study, and future research should be disseminated to the public and patients. Patient representatives felt it was important that during treatment clinicians should make patients aware that healthcare professionals may not “have all the answers” regarding exercise and there are gaps in the literature which require research. They felt this information would 1) help manage patient’s expectations 2) appreciate exercise requires tailoring and modifications before “getting it right” and 3) encourage other patients to take part in future research or PPI activities. As a result of our PPI, our dissemination activities will include 1) educating clinicians to discuss the degree of uncertainty regarding optimal exercise prescription when treating patients, to help manage expectations and encourage collaborative exercise prescription 2) updating our PPI literature to make it clear that there are uncertainties regarding exercise prescription requiring research that patients can be involved in.”* |
